# Metaphor Interpretation Using Paraphrases Extracted from the Web

**DOI:** 10.1371/journal.pone.0074304

**Published:** 2013-09-20

**Authors:** Danushka Bollegala, Ekaterina Shutova

**Affiliations:** 1 Department of Information and Communication Engineering, the University of Tokyo, Tokyo, Japan; 2 Institute for Cognitive and Brain Sciences, University of California, Berkeley, California, United States of America; University of Illinois-Chicago, United States of America

## Abstract

Interpreting metaphor is a hard but important problem in natural language processing that has numerous applications. One way to address this task is by finding a paraphrase that can replace the metaphorically used word in a given context. This approach has been previously implemented only within supervised frameworks, relying on manually constructed lexical resources, such as WordNet. In contrast, we present a fully unsupervised metaphor interpretation method that extracts literal paraphrases for metaphorical expressions from the Web. It achieves a precision of 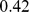, which is high for an unsupervised paraphrasing approach. Moreover, the method significantly outperforms both the baseline and the selectional preference-based method of Shutova employed in an unsupervised setting.

## Introduction

Metaphor is an important language tool that supports the creative nature of human thought and communication, enabling us to reason in novel, imaginative ways. Besides, it is a very common linguistic phenomenon manifested on average in every third sentence in general-domain text, according to corpus studies [2]. This makes computational processing of metaphor a pressing problem in NLP.

It has been previously shown that a number of real-world NLP applications could benefit from a metaphor processing component, e.g. machine translation [3], opinion mining [4], creative information retrieval [5] and recognizing textual entailment (RTE) [6]. Shutova [3] presents an example from machine translation (MT), where she studied the patterns of metaphor translation from English into Russian by the MT system Google Translate (http://translate.google.com/). She found that the MT system often produces literal translations of metaphorically used terms, rather than their literal interpretation, which makes the translated sentences semantically infelicitous in the target language. A metaphor processing component could help to avoid such errors. Ahmed [4] has shown that metaphor is often used when expressing strong opinions, which makes its automatic processing important for sentiment and opinion mining. Although existing Web information retrieval systems [7] can only search for literal matches of user queries, [5] proposes a figurative language retrieval model that can interpret metaphorical usage of language. Recognizing Textual Entailment (RTE), that involves recognizing whether one piece of text entails another is an important task in several natural language processing tasks such as question answering, text summarization and information extraction [8]. Agerri [6] shows that there is a significant correlation between the performance of textual entailment systems and their ability to interpret metaphorical expressions in texts.

Metaphors arise when one concept is viewed in terms of the properties of another. For example, consider the question *How can I* ***kill*** *a process?* [9]. Here, the *computational process* is viewed as being *alive* and therefore, its forced termination is perceived as *killing*. Metaphors can be explained via a systematic association, or a *mapping*, between two concepts or conceptual domains: the *source* and the *target* [10]. In our example, the *computational process*, which is the target concept, is viewed in terms of a *living being*, the source concept. The existence of such a mapping enables us to metaphorically describe the target domain using terminology borrowed from the source domain.

Several guidelines have been proposed in previous work to decide whether a particular word is used metaphorically or literally in a given context. For example, Shutoval et al. [2] annotate a verb as metaphorical if a more *basic* meaning of this verb can be established in a given context. As defined in the framework of MIP [11], basic meanings normally are: (1) more concrete; (2) related to bodily action; (3) more precise (as opposed to vague); and (4) historically order. Following [1], we define the task of metaphor interpretation as follows. Given a verb 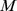, used metaphorically with a noun 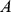, metaphor interpretation is the task of finding a non-metaphorical (i.e. literal) paraphrase 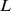 for 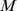 that expresses the same meaning as 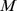 when used with 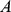. For example, to interpret the metaphorically used verb *kill* in the expression “*kill* a process” describing the noun *process*, one needs to extract the verbal paraphrase *terminate*.

Despite the vast potential applications of metaphor paraphrasing, it remains a challenging task for several reasons. Firstly, unlike many existing paraphrase extraction methods that derive paraphrases for nouns in isolation [12]–[18], we must identify paraphrases for the metaphorically used verb 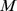 in the context of a noun 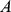. For example, although *assassinate* is a valid paraphrase for the verb *kill* from the point-of-view of traditional paraphrase extraction, it is not suitable for our purpose of interpreting the metaphorical phrase “*kill* a process” because the verb *assassinate* is not used with computer processes. Secondly, an extracted paraphrase for a metaphorical verb must be literal in order for it to be appropriate as an interpretation of the metaphorical verb. For example, consider the metaphorical expression “*reach* an agreement”. Although *arrive at* is a valid paraphrase for the verb *reach* in the traditional setting of paraphrase extraction, it is not suitable for the purpose of interpreting the metaphorical verb *reach* because “*arrive at* an agreement” is still a metaphorical expression. A better interpretation in this case would be *attain*.

Our method takes the above restrictions into account. Unlike previously proposed approaches for metaphor interpretation, it does not rely on manually compiled resources such as WordNet. Instead, it makes use of a Web search engine to generate a list of candidate paraphrases, and is thus fully unsupervised. The use of the Web for metaphor interpretation is beneficial for a number of reasons. First of all, this allows the method to find a larger number and a wider range of candidate interpretations, than a lexical resource-based method. In addition, it enables us to capture emerging novel and creative ways in which metaphors are used in the Internet, and can quickly adapt to change, as opposed to a method relying on static pre-compiled corpora.

**Figure 1:**
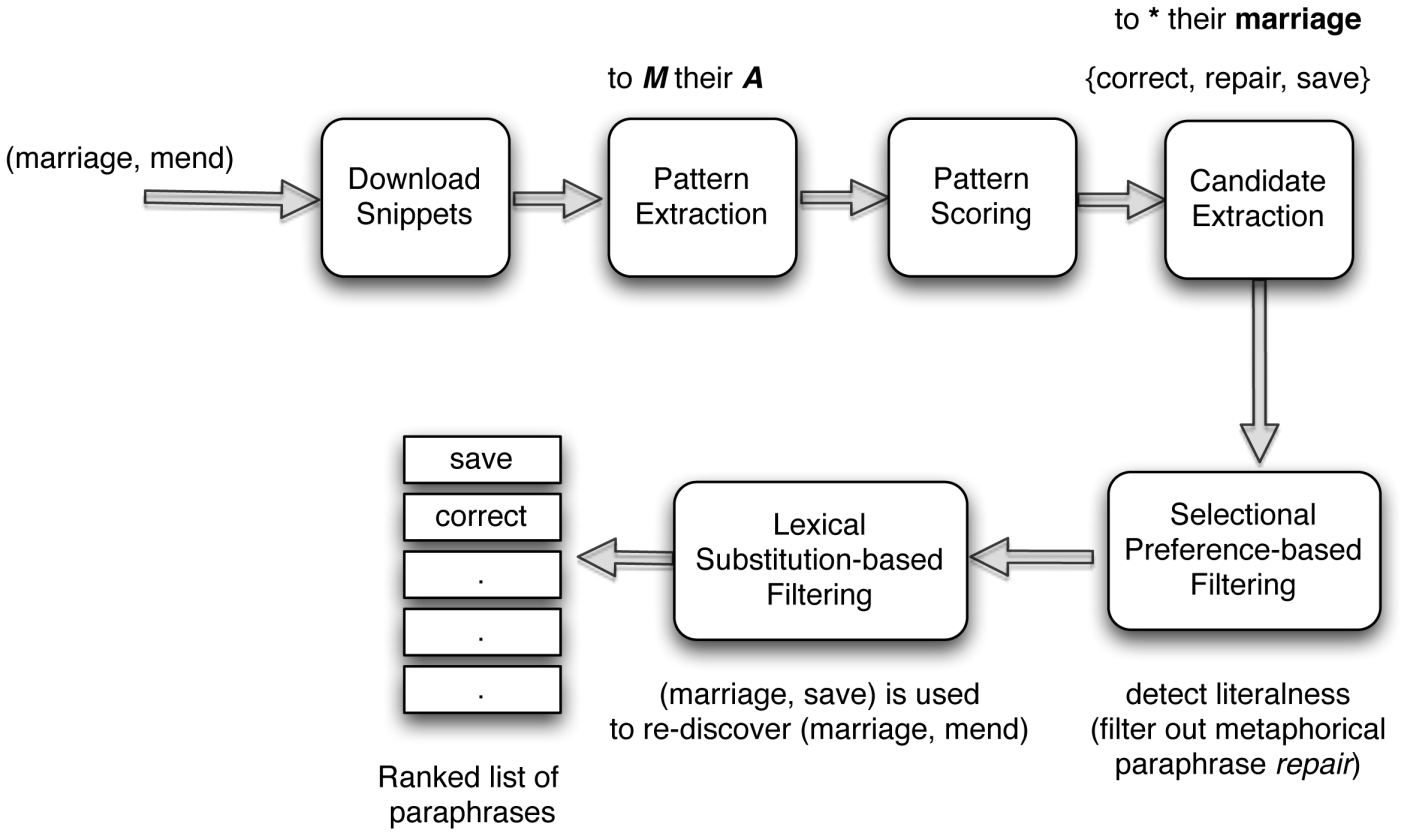
Outline of the proposed method. Given a noun and a metaphorical verb, we download snippets that contain the noun and the metaphorically used verb. Next, lexical patterns that represent the semantic relation between the noun and the verb are extracted and scored according to their representativeness. Then we use the top scored patterns to extract candidate paraphrases for the verb by searching for those patterns on the Web. Selectional preference is used to filter out metaphorical candidate verbs and a substitutability test is conducted to identify correct candidate paraphrases. Finally, a ranked list of non-metaphorical paraphrases for the original metaphorically used verb is returned.

Figure 1 illustrates the main components of our metaphor interpretation system. Given a metaphorical verb 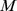 and its argument 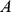, we first extract numerous lexical patterns from the Web to explicitly represent the semantic relation between 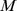 and 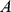. Lexical patterns are sequences of continuous words that are extracted from the local context of two words to represent the semantic relations that exist between those two words. For example, given the two words *ostrich* and *bird*, some of the lexical patterns that represent the semantic relation between those two words would be ***X*** *is a large* ***Y***, ***Y*** *s such as* ***X***, and *a large* ***Y*** *such as* ***X***. Here, we use ***X*** and ***Y*** respectively to denote the two words *ostrich* and *bird* in a lexical pattern. We use a pattern scoring method to select the highly representative lexical patterns for a particular semantic relation. For example, given the metaphorical expression “to *mend* a marriage”, one of the lexical patterns extracted by the proposed method is *to* ***M*** *their* ***A***, in which the placeholder variables ***M*** and ***A*** respectively denote the metaphorically used verb *mend* and its argument (object) *marriage*. We use bold italics to represent placeholder variables. Next, we query a Web search engine using the selected set of lexical patterns to find candidate paraphrases for the metaphorical expression. In our current example, some of the candidates we extract are: *correct*, *repair*, and *save*. Due to the noise in Web texts, there may be irrelevant paraphrases in the set of extracted candidates. Besides, some candidates may be used metaphorically again such as *repair*. To filter those out, we use a selectional preference-based model inspired by the work of Shutova [1]. In addition, we prioritize candidate paraphrases that have a high degree of lexical substitutability with the metaphorical word and show that this helps to avoid antonymous paraphrasing which is a common bottleneck in unsupervised lexical substitution. If a particular word can be substituted for another word in some context without altering the meaning of the context, then those two words are said to be lexically substitutable. Specifically, if a particular literal paraphrase 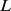 can be used to re-discover its metaphorical counterpart 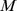 for a given argument 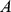, then such 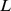 are considered to indicate higher meaning similarity and are ranked above other candidate paraphrases. Finally, a ranked list of candidates according to their appropriateness as literal paraphrases of the metaphorical verb in the given context (argument noun) is produced by the system.

We compare the performance of selectional preference and lexical substitutability-based models and evaluate them on verb–subject and verb–direct object constructions containing metaphorical verbs using the dataset of Shutova [1]. Our method achieves a precision score of 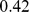, which is high for an unsupervised approach to lexical substitution. In particular, the proposed method significantly outperforms both a baseline method and the selectional preference-based method of [1] employed in an unsupervised setting. Moreover, the use of the Web enables us to discover paraphrases that are not listed in manually compiled resources for the metaphorical senses of verbs, which was one of the limitations of the approach of [1]. We also use a larger dataset of 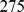 automatically extracted metaphorical expressions to further evaluate the proposed method for its scalability and robustness. Our proposed method outperforms two baselines in this evaluation demonstrating its applicability in a real-world metaphor interpretation system.

### Related Work

Because metaphor understanding requires drawing analogical comparisons, the development of a complete and computationally practical account of this complex phenomenon is challenging. The first approaches to metaphor identification and interpretation relied on manually-created knowledge-bases [19]–[22]. However, such approaches suffered from limited coverage, since manually created databases do not capture information about all possible domains and are expensive to build and extend. Two later approaches [1], [23] take a step away from metaphor-specific hand-coded knowledge and use corpora and lexical resources instead. [23] derive a “fluid knowledge representation for metaphor interpretation and generation” called Talking Points. Talking Points is a set of characteristics of concepts belonging to source and target domains and related facts about the world which are acquired automatically from WordNet and from the Web. Talking Points are organized in *Slipnet*, a framework that allows for a number of insertions, deletions and substitutions in definitions of such characteristics in order to establish a connection between the target and the source concepts. Consider the metaphor *Make-up is a Western burqa*:
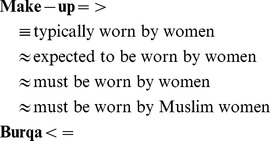



By doing insertions and substitutions the system arrives from the definition *typically worn by women* to that of *must be worn by Muslim women*, and thus establish a link between the concepts of *make-up* and *burqa*. Veale and Hao, however, did not evaluate to what extent their system is able to interpret metaphorical expressions in real-world text.

Shutova [1] defines metaphor interpretation as a paraphrasing task and presents a method for deriving literal paraphrases for metaphorical expressions from the British National Corpus (BNC) [24]. She first extracts a set of potential substitutes by selecting all words that appear in a particular syntactic relation with the metaphorical verb in the BNC. She then narrows down the list of candidates by selecting the verbs that share a hypernym with the metaphorical verb in WordNet. She uses automatically induced selectional preferences to discriminate between figurative and literal paraphrases. [1] reports a paraphrasing accuracy of 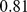. However, she uses WordNet for supervision, which limits the range of paraphrases that can be found by her method.

Shutova et al. [25] expanded on this work, addressing the metaphor paraphrasing task in an unsupervised setting. Their method first computes candidate paraphrases according to the context in which the metaphor appears, using a vector space model. It then uses a selectional preference model to measure the degree of literalness of the paraphrases. The authors evaluated their method on the metaphor paraphrasing dataset of [1] and report a top-rank precision of 0.52. The authors, however, point out that their method may suffer from data sparsity. Our approach aims to overcome this problem by performing web-based paraphrasing, that does not rely on pre-constructed corpora, but rather extracts a wide range of paraphrases from the web.

Shutova and Sun (2013) also addressed the problem of metaphor processing using unsupervised learning, however, only focusing on metaphor identification. Their method learns metaphorical associations from the data using graph-based hierarchical clustering of nouns. They report encouraging results for identification of metaphorical expressions in text (precision of 0.65), however, they do not apply their method to the problem of metaphor interpretation.

Extracting paraphrases from the Web has been studied in numerous tasks such as question answering [12], textual entailment recognition [26], relation extraction [13], and concept classification [27]. A common feature of these approaches is to repeatedly refine a set of candidate paraphrases using lexico-syntactic patterns in a bootstrapping process. However, as already explained in the Introduction section, our task of paraphrasing metaphorical expressions is different from the generic paraphrase extraction task in two important aspects. Firstly, we must extract literal paraphrases for metaphorically used words. The selectional preference-based filtering step constrains the candidate paraphrases based on their literalness. Secondly, we must select paraphrases for a metaphorical verb in a given context (it is only in context that a word can be used metaphorically). This is different from the generic paraphrase extraction setting in which a paraphrase extracted for a given phrase can replace it in all contexts. We impose this constraint by retaining the argument of the metaphorical verb in all lexical patterns during the candidate paraphrase extraction step. Despite those fundamental differences, existing paraphrase extraction systems might provide useful candidate paraphrases for metaphorical verbs that can be further filtered using the selectional-preference model and the lexical substitution component described in this paper. It will be an interesting future research direction for us to investigate those possibilities.

Given a metaphorical verb 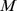 and its argument 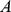, our metaphor interpretation method can be seen as finding a word 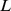 such that the *relational similarity* between the two word pairs 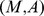 and 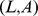 is high. Relational similarity between two word pairs is defined as the correspondence of the semantic relations (in our work represented as lexical patterns) that exist between the two words in each word pair [28]. Often, a high degree of relational similarity can be observed between analogous word pairs. The connection between analogy and metaphor has been pointed out in several previous works [19], [29]. For example, [19] in his met* algorithm shows that the presence of a relevant analogy is helpful to discriminate metaphorical relations from anomalous ones. [29] argues that the computational process used in understanding analogies to be the same as that used in understanding metaphors, and the difference is one of recognition and universality of acceptance in the underlying mappings.

Turney [30] proposed the dual space model for measuring the relational similarity between two pairs of words. Given two word pairs 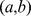 and 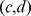, he uses lexical patterns that contain nouns to measure the *domain similarity* between two words, and lexical patterns that contain verbs to measure the *functional similarity* between two words. Then the relational similarity between two word pairs 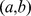 and 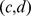 is computed using a combination of the functional and domain similarities of the words. Specifically, the relational similarity between the two word pairs 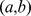 and 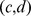 is computed as the geometric mean of the functional similarities between 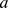 and 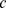 and 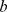 and 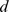, conditioned on the domain similarities of those words. Although relational similarity has so far been addressed as a task of relational classification, SemEval 2012 Task 2 [31] proposed a dataset that contains *degree* to which a certain word pair is representative of a particular semantic relation. They use the 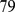 relational categories proposed by Bejar [32] and obtained relational similarity judgments using Amazon Mechanical Turk (www.mturk.com). Three systems participated in the evaluation task presenting six systems. However, only one of the systems was able to consistently outperform the baseline method that computed the degree of similarity using the pointwise mutual information between the two words in a word pair, which demonstrates the difficulty of the task. Although relational similarity has been successfully used in numerous tasks such as solving word analogy questions [28], [33], classifying noun-modifier relations [34], latent relational search [35], and recognizing synonyms, antonyms and associations [36], to our knowledge it has not yet been used for the task of interpreting metaphors.

## Methods

Our metaphor interpretation method operates in several steps: (1) extract lexical patterns to represent the semantic relations that exist between the metaphorical verb and its argument (noun), (2) use the extracted set of patterns to find candidate paraphrases for the metaphorical verb in the scope of its argument, (3) select literal paraphrases using a selectional preference model, and (4) perform a lexical substitutability test to recognize paraphrases that retain the original meaning of the metaphorical verb, thereby filter-out noisy extractions due to ambiguous lexical patterns, as well as antonymous paraphrases. The following subsections describe each of those steps in detail.

### Lexical Pattern Extraction

The first step towards metaphor interpretation is to identify the semantic relations that exist between a metaphorical verb and its argument. For example, consider the two sentences shown below.

1. Commentators claimed that she and Prince Charles had succeeded in *mending* their *marriage*.
2. After many hours doctors finally succeeded in *saving* their *patient*.

In (1) the verb *mend* is used metaphorically and takes *marriage* as its object. *Marriage* is viewed metaphorically as a machinery that can be *mended*. On the other hand, (2) provides an example of the verb *save* in its literal sense taking an actual human being (i.e. a *patient*) as its object. The lexical pattern *succeeded in* ***M*** *their* ***A*** occurs in both sentences between the verb 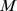 and its argument 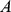, and acts as a mapping between the source and target concepts (i.e. *marriage* vs. *patient*). Lexical patterns have been successfully used in prior work to represent the semantic relations that exist between two words. These include, for example, IS-A relations [37], [38], verb relations [39], and entailment relations [40]. If we can find lexical patterns that describe the semantic relation between a metaphorical verb and its argument, then we can use those patterns to find potential paraphrases for the metaphorical verb.

Given a metaphorical verb, 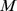, and its argument, 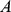, first we issue the conjugate query “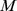 * * * 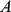
” to a web search engine. Here, the ‘*’ operator matches one or no words and is used to retrieve web pages in which both 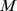 and 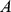 appear in close proximity, within a maximum of three words. The goal is to retrieve web pages that describe the semantic relationship between 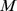 and 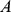. Moreover, the double quotation marks surrounding the two words ensure that the relative ordering of 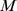 and 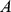 as specified in the query is also preserved in the search results. We download the top ranked search results returned by a Web search engine, and select sentences in which both 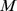 and 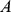 co-occur. We repeat this process with all inflectional forms of the metaphorical verb to increase the number of sentences we retrieve. Moreover, we reverse 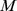 and 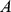 in queries to retrieve search results where 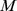 precedes 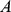 as well as 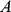 precedes 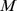. For example, for the phrase *mend marriage* the system issues queries such as “*mend* * * * *marriage*”, “*mending* * * * *marriage*”, “*mended* * * * *marriage*” etc.

We convert each selected sentence into lowercase and perform tokenization and lemmatization using Python Natural Language Toolkit (NLTK) (http://www.nltk.org). Next, we replace 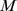 and 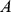 respectively with two placeholder variables ***M*** and ***A*** in each selected sentence. We then extract 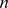-grams of word lemmas in each sentence such that each 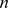-gram contains exactly one occurrence of ***M*** and one occurrence of ***A***. We vary 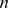 in the range 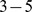 in our experiments. Those 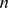-grams are then used as lexical patterns by the system in its subsequent processing. Unlike bag-of-words representations, 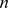-gram lexical patterns retain the relative ordering among words in a sentence. Moreover, most existing web search engines can be queried using 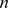-gram patterns, which is important when we use such patterns to find paraphrases for metaphorical verbs as we will describe later. Although extracting 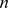-gram lexical patterns to represent semantic relations between two words using Web search engines has been previously frequently used for lexical acquisition from the Web, to our knowledge ours is the first attempt to apply this technique in the context of metaphor interpretation. We used the Google REST API (http://code.google.com/apis) in our experiments to search the Web. Table 1 illustrates an example of our lexical pattern extraction method.

**Table 1:** Extracting lexical patterns for the verb *mend* and its object *marriage*.

Query	“*mending* * * * *marriage*”
Sentence	Commentators claimed that she and Prince Charles had succeeded in *mending* their *marriage*
Lemmas	commentator claim that she and prince charles had succeed in ***M*** their ***A***.
Patterns	succeed in ***M*** their ***A***, in ***M*** their ***A***, ***M*** their ***A***

### Pattern Scoring

Not all extracted lexical patterns are equally representative of the semantic relation that exists between the two words in a word pair. Using a large set of marginally representative lexical patterns for extracting paraphrases often results in incorrect extractions because of the phenomenon known as the *semantic drift* [41], [42]. Moreover, using a large number of lexical patterns increases the number of web search engine queries required to extract paraphrases, thereby increasing the processing time. Therefore, we propose a pattern scoring method to efficiently select a small subset of lexical patterns that are highly related to the semantic relation that exists between the two words.

Let us consider a word 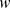 in a lexical pattern 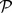 that is extracted for a word pair 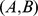. We define the relatedness, 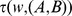, of 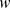 to the semantic relation implicitly described by 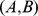 using pointwise mutual information, as follows,
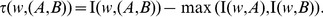
(1)


Here, 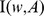, 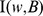, and 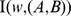 respectively denote the pointwise mutual information between 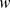 and word 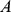, 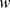 and word 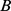, and 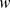 and word pair 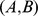. Relatedness is defined in Equation 1 as the difference between the two terms. The first term is the pointwise mutual information we gain about 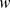 via the implicitly stated relation by the word pair 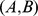. However, if 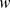 is highly correlated with only 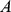 or 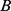, but not with the relation implied by the word pair 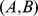, then we must discount patterns that contain such words 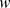. The second term in the right hand side of Equation 1 can be interpreted as the pointwise mutual information we gain about 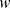 using only 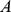 or 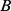. Because 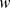 might be correlated with only 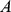 or 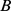, we consider the maximum of the two pointwise mutual information values instead of considering their average. Consequently, under Equation 1, words that describe the semantic relation that exists between the two words in a word pair obtain a higher score compared to words that are related to only 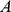 or 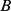.


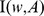
 is computed as follows:
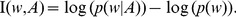
(2)


Here, 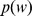 and 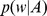 respectively denote the marginal probability of 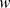, and the conditional probability of 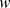 given 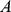. By substituting (2) in (1) we obtain
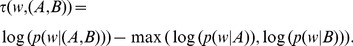
(3)


We approximate the conditional probability 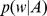 using the contexts we retrieve for 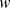 and 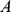 from the Web as follows,
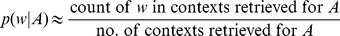
(4)


Likewise, we approximate 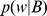 and 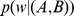 in Equation 3 using the counts of 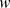 in contexts retrieved respectively for 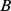 and the conjugate query *A AND B* to compute 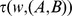. Finally, the score, 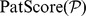, of a pattern 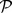 is computed as the sum of relatedness scores of all words 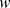 that appear in 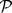 as follows,
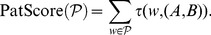
(5)


Although we experimented with a normalized version of the pattern scoring measure given in Equation 5 by dividing it from the number of words in a lexical pattern, this did not result in any significant improvement in the overall performance. Considering that we consider only 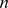-grams with 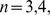 and 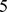 in our experiments (i.e. containing only 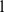, 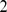, or 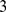 words in a pattern), we believe that normalization is not required for such short lexical patterns. Consequently, we use much simpler unnormalized version of the pattern scoring measure given in Equation 5.

It is noteworthy that the above-described pattern scoring method requires only three additional queries (i.e. 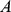, 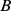 and *A AND B*) to the search engine to score all patterns extracted for a word pair 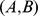. In particular, we do not require any queries that involve 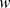 during pattern scoring. This enables us to efficiently score a large number of lexical patterns using fewer Web queries, thereby minimizing the load on the search engine. In addition, this pattern scoring method does not rely on page counts (or Web hits), as it was the case in most previous work on Web-based word association measures. This is an advantage, because the Web hits are known to be unreliable approximate counts [43], [44].

### Candidate Paraphrase Extraction

To extract candidate paraphrases for a metaphorical verb 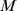, we construct search queries using the lexical patterns extracted and scored as described above. Specifically, for a lexical pattern extracted for a word pair 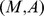, we replace ***M*** by a wildcard “*”, and ***A*** by the argument 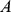. For example, for the lexical pattern *succeed in* ***M*** *their* ***A*** shown in Table 1, we construct the query suceed in * their marriage. The wildcard will match at most one word in a web document. We then retrieve web documents that contain those lexical patterns and match each individual pattern separately as a regular expression to find the words that match the slot corresponding to the wildcard in each search result.

However, not all words extracted by this procedure are valid paraphrases. Firstly, given the noise in web data, a pattern might match texts that produce irrelevant candidates. Secondly, a single pattern might not sufficiently represent the semantic relation between 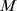 and 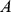. Therefore, extracting candidates only by a single lexical pattern is unreliable. To overcome those problems we propose a candidate scoring method that considers all lexical patterns collectively for selecting the most relevant candidate paraphrases. Specifically, we consider both the number of times a candidate paraphrase 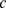 is extracted by a particular lexical pattern 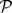 (denoted by 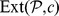), and the score assigned to the pattern 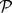 (Equation 5). We sum the product of those two factors to compute the score, 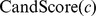, of a candidate 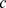 as a paraphrase for a metaphorical verb 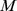 as follows,
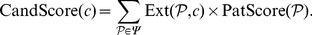
(6)


Here, 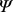 denotes the set of lexical patterns extracted for the word pair 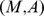 by the pattern extraction method. We rank the extracted set of candidates in the descending order of their scores, and select the top 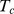 candidates for further processing. According to the candidate scoring Formula 6, candidates that are extracted numerous times by high scoring patterns (scored using Formula 5) will receive high scores and are preferred as interpretations of the metaphorical verb.

### Selectional Preference-based Filtering

Following [1], we use a selectional preference model to discriminate between literally and metaphorically used candidate substitutes. For example, for the metaphorical expression “*accelerate* change” the system extracts a metaphorical paraphrase “*catalyse* change”, as well as a literal one “facilitate change”. Verbs used metaphorically are likely to demonstrate semantic preference for the source domain, e.g. *catalyse* would select for chemical reactions, rather than change (the target domain), whereas the ones used literally for the target domain, e.g. *facilitate* would select for processes (including change). We therefore expect that selecting the verbs whose preferences the noun in the metaphorical expression matches best should allow us to filter out non-literalness.

We replicated Shutova’s method and automatically acquired selectional preference (SP) distributions of the candidate substitutes (for subject-verb and verb-object relations) from the BNC parsed by the RASP parser [45]. We obtained SP classes by clustering the 2000 most frequent nouns in the BNC into 200 clusters using the algorithm of [46]. We quantified selectional preferences using the association measure proposed by [47]. It represents SPs as the difference between the posterior distribution of noun classes in a particular relation with the verb and their prior distribution in that syntactic position irrespective of the identity of the verb. This difference then defines the *selectional preference strength* (SPS), 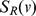, of the verb, 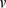, quantified in terms of Kullback-Leibler divergence as follows
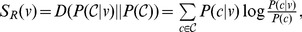
(7)where 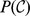 is the prior probability of the noun class 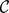, 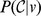 is the posterior probability of the noun class 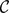 given the verb 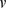 and 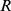 is the grammatical relation. For each noun 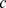 in the noun class 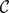, we consider the sum of the terms involving the conditional probability 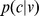 and the prior probability 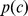 as shown in Equation 7. SPS measures how strongly the predicate constrains its arguments [40], [48]. Resnik then quantifies how well a particular argument class 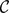 fits the verb 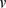 using another measure called *selectional association*:



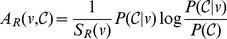
(8)We use selectional association as a measure of semantic fitness, i.e. literalness, of the paraphrases. The candidate paraphrases were re-ranked based on their selectional association with the class of the noun in the context. Those paraphrases that are not well suited or used metaphorically are dispreferred within this ranking. The system then selects the top 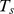 paraphrases as ranked by this method for further processing.

### Lexical Substitutability

Given a metaphorical verb 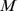 and its argument (noun) 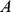, in previous section we described a method to represent the word pair 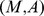 using a set of lexical patterns. Each extracted lexical pattern can be considered as representing some context in which the metaphorical verb 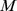 co-occurs with its argument 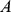 in the Web. We then measured the appropriateness of a candidate 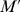 as a paraphrase for 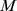 by considering the co-occurrence of 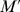 with 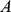 in the set of lexical patterns we extracted for the word pair 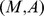. This distributional approach for extracting paraphrases is based on the distributional hypothesis [49], [50] and works as follows – if 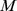 and 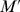 co-occur with 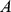 in common lexical patterns then the likelihood of 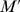 as a paraphrase of 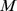 increases. Although distributional similarity has been successfully used in numerous previous work to extract synonyms [51], related words [52], or paraphrases [13], [53], it is known to extract antonyms which are also highly distributionally similar [54]. Unfortunately, the selectional preference-based filter is focused on detecting literalness and would not remove the antonymous paraphrases.

A popular solution advocated in existing paraphrase extraction systems to the antonymy problem is to use bilingual dictionaries or parallel corpora and filter-out paraphrases that do not correspond to the same target in the multiple languages [14]–[18], [55]. This approach works well in practice because although two words might be antonyms in one language, their translations are often non-antonymous in another language [56]–[58]. However, in our setting, unsupervised metaphor interpretation, we do not assume the availability of bilingual lexical resources or parallel corpora and cannot apply this solution.

Instead, we propose a lexical substitutability [59] test that is based on the observation that the sentential contexts in which two antonyms occur differ each other to the extent that antonyms are not readily substitutable for one another [57], [58]. In other words, antonymy is a lexical association between word pairs, and antonymous words do not follow the substitutability hypothesis [56], [57]. On the other hand, synonymy is a symmetric semantic relation – it must be possible to substitute 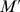 in place of 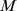 in the contexts in which 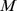 and 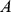 co-occurs. If we can start with 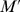 as the verb and repeat the above process to discover 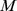, then the reliability of 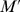 as a paraphrase of 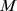 can be considered to be high.

Specifically, we use each paraphrase 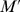 with the argument 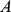 of the metaphorical verb 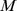 to form a word pair 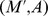, and use the pattern extraction method described in the previous section to extract a set of lexical patterns that represents the semantic relations between 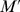 and 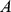. Next, we use the pattern scoring method to identify the most relevant lexical patterns for the semantic relation between 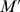 and 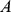, and use those lexical patterns to extract candidates. If 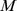 can be retrieved using 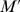, then we select such candidates 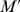 as potential paraphrases for 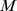. Otherwise, the candidates are removed. Moreover, we re-rank the selected candidates 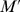 by the candidate score of 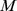 (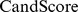). For example, let us assume that 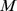 receives a 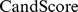 of 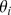 when the paraphrase 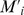 is used, then we rank the paraphrases 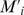 in the descending order of the corresponding 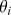 values.

## Experiments

### Datasets

We use the dataset of [1], who annotated metaphorical expressions in a subset of the BNC sampling text from various genres. This dataset consists of 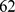 subject-verb and verb-object constructions, where a verb is used metaphorically. The expressions in the dataset include e.g. *stir excitement, reflect enthusiasm, accelerate change, grasp theory, cast doubt, suppress memory, throw remark* (verb-object constructions) and *campaign surged, factor shaped, tension mounted, ideology embraces, changes operated, approach focuses, example illustrates* (subject-verb constructions). 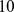 phrases in the dataset were used for development purposes, and the remaining 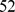 constituted the test set. To our knowledge, this is the only metaphor paraphrasing dataset and gold standard available to date. In addition, it allows us to directly compare our results to the work of [1].

In addition to the evaluation against this small, manually-annotated benchmark dataset, we also evaluate our system on a larger automatically created dataset. This dataset was created using the state-of-the-art metaphor identification system of Shutova et al. [60]. This system identifies verb-object and verb-subject metaphorical expressions in a large corpus. It starts from a small set of seed metaphors and then learns patterns of the use of metaphor by means of co-clustering of verbs and nouns. We ran the pre-trained system of Shutova et al. [60] on the BNC and extracted a number of metaphorical expressions from the corpus. We then randomly selected a set of metaphorical expressions from the output of the system and manually filtered out the ones that were ungrammatical due to parser errors. This resulted in 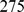 metaphorical expressions that constitute our second evaluation dataset. We then applied our method to generate and rank candidate paraphrases for the metaphorically used verbs. We manually labeled each extracted candidate paraphrase indicating whether it is a literal paraphrase for the metaphorical verb or not and evaluated the system against these annotations.

The small dataset of manually annotated metaphorical expressions of [1] contains more accurate annotations than the automatically created one, which may contain a certain degree of noise. It also allows us to directly compare our method to previous approaches to this task. However, the evaluation on the automatically created dataset is larger in scale, as well as it allows us to see how applicable the proposed method is for real-world tasks (often dealing with noisy data) and external NLP applications that can benefit from the use of integrated metaphor processing (i.e. a combination of metaphor identification and interpretation within a single system). To enable other researchers to reproduce our results in the future we make both the source code and the crawled data publicly available (http://www.iba.t.u-tokyo.ac.jp/~danushka/data/MetaAna.tgz).

### Baseline and Systems

We compare the paraphrases produced by our method at two different stages against a Web-based baseline.

#### Baseline

We use the top 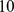 candidate paraphrases produced by the candidate extraction step and ranked in the descending order of their 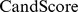, as a baseline. This choice of baseline highlights the effect of using a selectional preference model and lexical substitutability to identify literal paraphrases for metaphorical expressions.

#### SP

[1] proposed the use of selectional preference to identify literal paraphrases for metaphorical phrases. We compute selectional preference scores as described earlier for the candidates extracted by the proposed method and rank those candidates in the descending order of their selectional association scores. This method demonstrates the level of performance we obtain if we do not use the lexical substitutability-based paraphrase re-ranking. This method can be regarded as an unsupervised variant of Shutova’s supervised metaphor interpretation method [1], in which the candidate paraphrases are selected not from the WordNet synsets but from the Web.

#### SP-LexSub

For a metaphorical phrase, we extract candidate paraphrases and select top 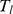 candidate paraphrases based on their selectional association scores. We then use the lexical substitutability method to induce a relative ordering among those candidates and filter out irrelevant candidates. We set the values of 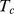, 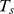, and 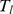 experimentally using the development portion of the dataset. Specifically, we measure the precision at rank 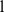 (described in the next Section) for the metaphorical expressions in the development dataset and set the values 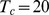, 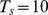, and 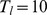 such that the average precision at rank 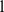 is maximized. The remainder of the experiments described in the paper are conducted with those parameter values.

### Evaluation Methods

We evaluate the paraphrases produced by the three systems with the aid of human judges, and against a human-created gold standard in two different experimental settings.

#### Setting 1

Two independent human judges were presented with a set of sentences containing metaphorical expressions and their rank 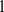 paraphrases produced by the three methods, randomized. Both judges were native speakers of English and had linguistics background. They were asked to mark the ones that have the same meaning as the metaphorically used term and are used literally in the context of the paraphrase expression as correct.

We then evaluate the system’s performance against their judgements in terms of precision at rank 1, 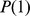. Precision at rank 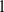 measures the proportion of correct literal interpretations among the paraphrases in rank 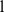. A paraphrase was considered correct if both judges marked it as correct. The inter-judge agreement for this evaluation was measured at 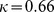, which is considered substantial.

#### Setting 2

We then also evaluate the system and baseline rankings against a human-constructed paraphrasing gold standard of [1]. Shutova asked five annotators (native English speakers) to write down all suitable literal paraphrases for the highlighted metaphorical verbs in a set of sentences. The gold standard was then compiled by incorporating all of their annotations. For example, the gold standard for the phrase *brushed aside the accusations* contains the verbs *rejected, ignored, disregarded, dismissed, overlooked*, and *discarded*.

However, it should be noted that given that metaphor paraphrasing task is open-ended, it is hard to construct a comprehensive gold standard. For example, for the phrase *stir excitement* the gold standard includes the paraphrase *create excitement*, but not *provoke excitement* or *stimulate excitement*, which are more precise paraphrases. Thus the gold standard evaluation may unfairly penalize the system, which motivates our two-phase evaluation against both the gold standard and direct judgements of system output. A post-hoc solution would be to append all the paraphrases marked by the human judges in **Setting 1** as correct to the gold standard dataset, thereby improving the coverage of the gold standard. However, we decided against this post-hoc solution because it would make it difficult to interpret our results against previously proposed results using this gold standard dataset.

Following [1], the system output is compared against the gold standard using *mean reciprocal rank* (MRR) [7] as a measure. MRR assess ranking quality beyond rank 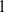 and is defined as follows:
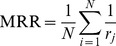
(9)where 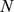 is the number of metaphorical expressions in the human gold standard dataset, 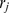 is the rank of the first correct literal interpretation (according to the human gold standard) found among the top five paraphrases.

## Results and Discussion

We compare the performance of the **SP-LexSub** method against **SP** and **Baseline** on Verb-DirectedObject and Verb-Subject relations separately, as well as across the whole dataset. Tables 2 and 3 show the experimental results in terms of system precision at rank 1 (Setting 1) and MRR (Setting 2) respectively. Results in both settings demonstrate that the **SP-LexSub** method outperforms both **Baseline** and **SP** for the Verb-DirectObject relation, as well as Verb-Subject. In particular, the improvements shown by the **SP-LexSub** method against the **Baseline** and the **SP** methods in Table 3 are statistically significant under paired t-test (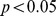).

**Table 2:** Precision at rank 1 for different methods measured against human judgements.

Relation	Baseline	SP	SP-LexSub
Verb-DirectObject	0.33	0.28	0.44
Verb-Subject	0.14	0.14	0.29
Across dataset	0.30	0.26	0.42

**Table 3:** Comparison of different methods against the gold standard using MRR.

Relation	Baseline	SP	SP-LexSub
Verb-DirectObject	0.122	0.217	0.265
Verb-Subject	0.088	0.166	0.219
Across dataset	0.115	0.206	0.256

**SP** shows a slightly lower performance than the **Baseline** method in Setting 1, as opposed to Setting 2. Such a discrepancy can be explained by the fact that 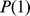 is oblivious of the overall ranking beyond rank 1 and the recall of paraphrases, whereas MRR takes this into account. This suggests that the **SP** method does outperform the baseline overall and emphasizes correct paraphrases, while de-emphasizing the incorrect ones. Note that, although 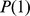 is never greater than MRR for the same list of ranked items, this property does not hold for the numbers shown in Tables 2 and 3 because the experimental settings are different (human judgements vs. comparison against gold standard dataset) in the two evaluations.

Performance of the proposed method and the baselines on the automatically collected larger dataset of 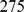 metaphorical expressions is shown in Table 4. From Table 4, we see that the proposed **SP-LexSub** method outperforms the **Baseline** method and the **SP** method even in this larger dataset of metaphorical expressions. This result shows the robustness of the proposed metaphor interpretation method in handling automatically detected metaphorical expressions over a larger dataset.

**Table 4:** Comparison of the different methods on the automatically collected metaphorical expressions using MRR.

Method	MRR
**Baseline**	0.436
**SP**	0.488
**SP-LexSub**	**0.526**

The errors of the **SP** method were concentrated around the presence of a large number of antonymous paraphrases provided by the initial candidate extraction (e.g. “waive a decision” for “*impose* a decision”). Since the selectional preference model is suited to detect literalness rather than meaning retention, antonymous paraphrases that have a high semantic fit into the context may get ranked equally high. Hence, as we expected, additional processing is needed to filter out antonymous and irrelevant candidates, as performed by the **SP-LexSub** method. The results confirm this and **SP-LexSub** achieves the highest performance both in terms of 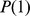 and MRR for both types of constructions, as well as across the dataset. Example paraphrases produced by the method include “forget the past” for “*disown* the past”, “formulate a theory” for “*develop* a theory” and “raise doubt” for “*cast* doubt”. Overall, all methods show better results for the Verb-DirectObject relation than the Verb-Subject relation. However, there are only 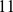 Verb-Subject metaphorical expressions, as opposed to the 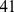 Verb-DirectObject ones in the test set. Therefore, a larger dataset that contains more Verb-Subject metaphorical expressions is required to further analyze this trend.

Table 5 shows an example of the paraphrase rankings produced by the three methods (scores shown in brackets) for the word pair (*impose*, *decision*). The correct literal paraphrase for the metaphorical verb according to the gold standard (*enforce*) is shown in italics. One can see that **SP-LexSub** ranks the correct paraphrase at the first rank, whereas the **Baseline** does not list the correct paraphrase among the top 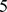. Moreover, the antonyms of *impose* such as *waive* and *lift* are also extracted and ranked at the top by the **Baseline**. However, the lexical substitutability constraint successfully eliminates such antonyms, improving the performance of the system.

**Table 5:** Top 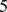 paraphrases ranked for the word pair (*impose*, *decision*) with their scores.

Baseline	SP	SP-LexSub
waive ( 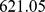 )	uphold ( 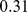 )	*enforce* ( 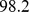 )
lift ( 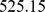 )	revoke ( 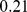 )	delay ( 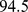 )
ease ( 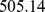 )	*enforce* ( 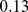 )	implement ( 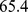 )
apply ( 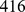 )	implement ( 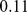 )	uphold ( 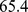 )
award ( 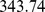 )	postpone ( 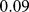 )	reinforce ( 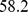 )

The error analysis has shown that most errors of the system result from metaphorical paraphrasing (e.g. “*illuminate* aspects” for “*illustrate* aspects”), imprecise paraphrasing (e.g. “publish a report” for “*leak* a report”) or sometimes still antonymous paraphrasing (e.g. “address subject” for “*overlook* subject”). Cases where the top-ranked paraphrases were entirely unrelated (e.g. “redefine a problem” for “*confront* a problem”) are rare (13%).

Our results are lower than those of the supervised WordNet-based method of Shutova [1], who achieved 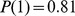 and 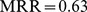. However, our results are in line with the performance of other unsupervised lexical substitution methods, whose accuracy tends to be lower than that of the supervised ones, for example Shutoval et al. [25] reports a Mean Average Precision (MAP) score of 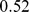. We have shown that the selectional preference-based ranking of Shutova [1] designed to detect literalness of the paraphrases is less applicable in an unsupervised setting, where the problem of antonymous paraphrasing is more common. We successfully addressed this problem by estimating the degree of lexical substitutability of the paraphrases in addition to their literalness, which significantly improved the overall system performance.

## Conclusions

We presented an unsupervised metaphor interpretation method that uses the Web to find literal paraphrases for metaphorical expressions. The method discovers an extensive number of potential candidates, yielding high recall. At the same time, the use of literalness (SP) and meaning retention (LexSub) filters allows it to achieve an encouraging level of precision for an unsupervised approach. We showed that the selectional preference-based ranking of [1] designed to detect literalness of the paraphrases is less applicable in an unsupervised setting, where the problem of antonymous paraphrasing is more common. We successfully addressed this problem by applying the lexical substitutability filter in addition to the SP literalness filter, which significantly improved the overall system performance. Using automatically extracted lexical patterns to query a Web search engine allows the method to discover an extensive number of potential candidates, yielding high recall. At the same time, the use of literalness (SP) and meaning retention (LexSub) filters allows it to achieve a precision of 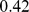, which is an encouraging result in unsupervised lexical substitution. Our future plans include extending the system to process further syntactic constructions, as well as is to build large scale metaphor gold standards for different parts of speech by crowd sourcing.
